# Idiopathic pulmonary fibrosis: Current and future treatment

**DOI:** 10.1111/crj.13466

**Published:** 2022-01-10

**Authors:** Daniel S. Glass, David Grossfeld, Heather A. Renna, Priya Agarwala, Peter Spiegler, Joshua DeLeon, Allison B. Reiss

**Affiliations:** ^1^ Department of Medicine and Biomedical Research Institute NYU Long Island School of Medicine Mineola New York USA

**Keywords:** idiopathic pulmonary fibrosis, lung transplantation, nintedanib, pentraxin, pirfenidone

## Abstract

**Objectives:**

Idiopathic pulmonary fibrosis (IPF) is a chronic fibrotic lung disease characterized by dry cough, fatigue, and progressive exertional dyspnea. Lung parenchyma and architecture is destroyed, compliance is lost, and gas exchange is compromised in this debilitating condition that leads inexorably to respiratory failure and death within 3–5 years of diagnosis. This review discusses treatment approaches to IPF in current use and those that appear promising for future development.

**Data Source:**

The data were obtained from the Randomized Controlled Trials and scientific studies published in English literature. We used search terms related to IPF, antifibrotic treatment, lung transplant, and management.

**Results:**

Etiopathogenesis of IPF is not fully understood, and treatment options are limited. Pathological features of IPF include extracellular matrix remodeling, fibroblast activation and proliferation, immune dysregulation, cell senescence, and presence of aberrant basaloid cells. The mainstay therapies are the oral antifibrotic drugs pirfenidone and nintedanib, which can improve quality of life, attenuate symptoms, and slow disease progression. Unilateral or bilateral lung transplantation is the only treatment for IPF shown to increase life expectancy.

**Conclusion:**

Clearly, there is an unmet need for accelerated research into IPF mechanisms so that progress can be made in therapeutics toward the goals of increasing life expectancy, alleviating symptoms, and improving well‐being.

## 1 INTRODUCTION

Idiopathic pulmonary fibrosis (IPF) is a chronic interstitial lung disease characterized by fibrosis, inflammation, and destruction of lung architecture.^1^, ^2^, ^3^ Damage to the alveolar epithelium and abnormal wound repair are theorized to be key factors in the development of this disease. IPF is thus far incurable with average onset at about age 65 years and a survival rate of 3–5 years after diagnosis. Likelihood of living 5 years from time of diagnosis ranges from 20% to 40%.^4^ About 70% of patients are male and history of cigarette smoking is common, with the number of IPF patients estimated at 5.6 per 100 000 per year.^5^, ^6^, ^7^ The causes of IPF remain unknown, although it is thought to result from a combination of genetic and environmental factors. Constant micro‐injuries to aging alveolar epithelium are believed to lead to disrupted epithelial–fibroblast communication, which culminates in recruitment and activation of myofibroblasts that produce collagen‐rich extracellular matrix. Excessive accumulation of this matrix renders alveoli irreversibly collapsed and nonfunctional, and the result is reduced gas exchange and difficulty breathing.^8^, ^9^ Since there is no cure, treatment has remained focused on slowing progression of fibrosis, maintaining comfort and, in late stages, on palliative care.^5^, ^6^, ^10^, ^11^, ^12^, ^13^ Eventually, IPF patients die from respiratory failure, often during an acute exacerbation or from the effects of another comorbidity such as cardiovascular disease, lung cancer, or thromboembolism.^14^, ^15^, ^16^ The lack of curative treatment demands aggressive pursuit of better choices as described throughout this review.

## 2 PRESENTATION AND DIAGNOSIS

Early diagnosis of IPF is difficult because initial symptoms, most commonly dyspnea and cough, are mild and nonspecific and overlap many other common conditions.^17^, ^18^, ^19^ Once symptoms become more pronounced, the diagnosis can be made and antifibrotic therapy options may be initiated.^20^ Upon physical examination, respiratory auscultation may detect Velcro crackles and clubbing of fingers may be apparent.^21^, ^22^, ^23^ Pulmonary function testing (PFT) shows a restrictive pattern measured as reduced forced vital capacity (FVC), reduced forced expiratory volume in 1 s (FEV_1_), and reduced efficiency of lung gas transfer estimated with measurement of the diffusion capacity of carbon monoxide (DL_CO_). The Gender, Age, and Physiology (GAP) Index Score encompasses both FVC and DL_CO_ along with age and gender and is considered a practical index and staging system for predicting IPF mortality.^24^

The 6‐min walk test, an easy and straightforward way to determine exercise capacity by having the patient walk as far as possible in 6 min on a flat surface, has prognostic value since faster decline in distance covered is associated with higher mortality.^25^, ^26^

**FIGURE 1:**
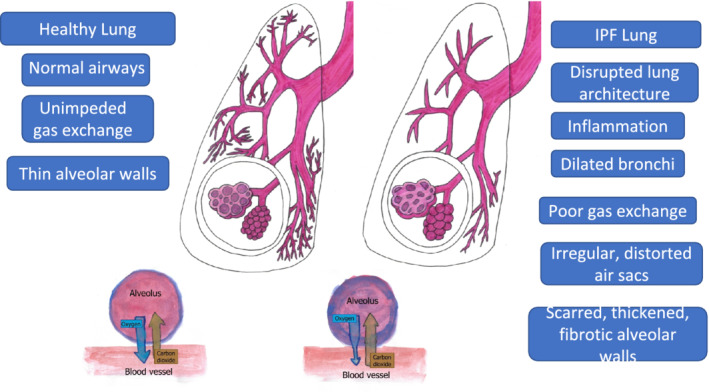
Comparison of healthy lung to idiopathic pulmonary fibrosis (IPF) lung. The healthy lung is characterized by unscarred airways with thin‐walled alveoli and unimpeded gas exchange. Pathological features of the IPF lung include dilated bronchi, airway distortion, and thickened alveolar walls. Inflammation and fibrosis lead to impaired gas exchange within the alveoli

The histopathological features of IPF are those of usual interstitial pneumonia (UIP) which appears under the microscope as a heterogeneous patchwork of focal areas of fibrosis with hyperplastic alveolar epithelial cells adjacent to these fibroblastic foci, alternating with less affected areas of normal or nearly normal lung tissue.^27^ Lung architecture is disrupted leading to impaired gas exchange (Figure 1).^6^

IPF is most often imaged by computer tomography (CT), but a role for magnetic resonance imaging is emerging.^28^, ^29^ The most typical radiological pattern is UIP.^30^ Extent of fibrosis on CT can be assessed visually or quantified more objectively using computer algorithms.^31^, ^32^ High‐resolution CT often shows honeycomb changes, traction bronchiectasis, and a reticular pattern that is predominantly in the periphery of the lower lobes.^5^, ^33^, ^34^, ^35^

In addition to the poor prognosis, IPF patients often have multiple comorbidities including hypertension, chronic obstructive pulmonary disease (COPD) emphysema, diabetes mellitus, and gastroesophageal reflux disease (GERD).^36^, ^37^ These can decrease quality of life, complicate treatment, increase hospitalization, and contribute to deterioration of the patient's health.

## 3 PAST TREATMENT STRATEGIES TARGETING INFLAMMATION

Until 2014, the standard practice for treating IPF focused primarily on immunosuppressant therapy using a combination of prednisone, azathioprine, and *N*‐acetylcysteine.^5^ However, after the release of the results from the Evaluating the Effectiveness of Prednisone, Azathioprine and N‐acetylcysteine in Patients with IPF (PANTHER‐IPF) TRIAL in 2012, it became apparent that, compared with placebo, this form of treatment actually increased the likelihood of hospitalization, treatment‐related severe adverse events, and death in IPF patients.^38^ In IPF subjects, a post hoc analysis across PANTHER‐IPF and a second large multisite clinical trial (ACE‐IPF AntiCoagulant Effectiveness in IPF [ACE‐IPF]) found an inverse association between leukocyte telomere length and poor outcome in response to immunosuppressants.^39^ Immunosuppressants such as cyclophosphamide and mycophenolate showed no benefit in IPF clinical trials and are now considered inadvisable for IPF while steroids are still used to reduce cough.^40^, ^41^, ^42^, ^43^

Thalidomide is a drug with an infamous history, originally intended for use as a sedative and an anti‐emetic; it caused serious teratogenic effects and was subsequently removed from the market.^44^ However, since then, thalidomide has been found to be an effective treatment for several cancers, such as multiple myeloma and kidney cancer, as well as inflammation‐based diseases.^45^, ^46^ The anti‐inflammatory properties of thalidomide are what make this glutamic acid derivative a compelling contender for treating IPF, and there has been speculation about its properties being used for such a purpose for many years.^47^ However, the information and amount of testing done to explore the potential utility of thalidomide in IPF is still very limited.

In 2007, thalidomide was used on bleomycin‐induced pulmonary fibrosis in mice and in 2015 it was shown to protect against emphysematous changes in mice exposed to cigarette smoke.^48^, ^49^ In the bleomycin model, C57BL/6 mice received intraperitoneal bleomycin sulfate with and without intervention with intraperitoneal thalidomide. Those given thalidomide showed reduced collagen deposition in the lung and accompanying this histologic benefit, thalidomide mitigated bleomycin‐induced upregulation of interleukin‐6 and transforming growth factor (TGF)‐β.

In 2012 at Johns Hopkins University School of Medicine, a 24‐week double blind randomized trial evaluated the antitussive action of thalidomide in IPF.^50^ In this study, 24 IPF patients were randomly assigned to receive 12 weeks of thalidomide or placebo followed by a 2‐week washout and then a crossover for a further 12 weeks. While receiving thalidomide, patients showed a definite increase in comfort and quality of life as measured by the Cough Quality of Life Questionnaire. However, it was a very small study and only 20 patients completed the trial. Subsequently, a panel of experts did not recommend thalidomide treatment for cough in IPF.^51^

In 2017, a cell culture study using rat lung epithelial cells (CCL‐149) showed that the presence of thalidomide mitigated TGF‐β1‐stimulated epithelial to mesenchymal transition.^52^ Despite documented anti‐inflammatory properties of thalidomide, there have been no significant further attempts to study its effect on IPF in human trials. This lack of momentum and enthusiasm likely reflects long‐held deeply negative associations.

## 4 CURRENT PHARMACOLOGIC TREATMENTS

Two antifibrotic therapies have been approved for the treatment of IPF: pirfenidone and nintedanib (Table 1). Pirfenidone is a modified pyridine small molecule with antifibrotic, anti‐inflammatory and antioxidant properties. It decreases the production of collagen, slows the fibrotic process by suppressing the cytokine TGF‐β and lowers the rate of decline in FVC.^53^, ^54^, ^55^ This was shown in two phase 3 CAPACITY trials and the ASCEND trial.^56^, ^57^, ^58^ Pirfenidone lowers the likelihood of respiratory‐related hospitalization over the course of 1 year of treatment.^59^ A retrospective study of 34 IPF patients in the North of England who were treated with perfenidone found greater benefit to those patients who were experiencing more rapid decline (≥5% decrease in FVC per year) prior to administration of the drug.^60^ While pirfenidone is generally well‐tolerated and the side effects are mild (skin rash, weight loss, nausea, and fatigue), there have been cases of abnormal liver function, particularly elevation of serum alanine aminotransferase (ALT)/aspartate aminotransferase (AST) and bilirubin and therefore patients being treated with pirfenidone need regular monitoring of liver function.^61^, ^62^, ^63^

**TABLE 1:** IPF treatments

Treatment	Mechanism of action	Clinical effects
Current therapies		
Pirfenidone	Antifibrotic and anti‐inflammatory	Slows rate of decline in FVC
Nintedanib	Antifibrotic and anti‐inflammatory	Slows rate of decline in FVC
Oral corticosteroids, opioids	Antitussive	Decrease cough—improve quality of life
Anti‐acids, proton pump inhibitors	Reduces GERD	Benefits unclear
Lung transplantation	Surgical replacement of one lung or both lungs	Only available potentially curative therapy
Therapies in development		
PRM‐151	Recombinant human pentraxin‐2; acts as an antifibrotic agent	Slows rate of decline in FVC
Pamrevlumab	Fully human recombinant monoclonal antibody against CTGF	Slows rate of decline in FVC
TD139	Small molecule inhibitor of galectin‐3	Decreases plasma biomarkers of inflammation. Study in progress to asses effect on FVC
PLN‐74809	Blocks activation of the TGFβ pathway	Study in progress with end‐points of safety, tolerability, pharmacokinetics
TRK‐250	Suppresses expression of TGFβ	Study in progress to assess safety and tolerability of single and multiple inhaled doses

Abbreviations: CTGF, connective tissue growth factor; FVC, forced vital capacity; GERD, gastroesophageal reflux disease; IPF, idiopathic pulmonary fibrosis.

Nintedanib is an intracellular tyrosine kinase inhibitor that binds to adenosine triphosphate binding sites, thus suppressing the signaling pathways linked to vascular endothelial growth factor receptor, fibroblast growth factor receptor 1–3, and platelet‐derived growth factor receptor α and β. These effects on receptor tyrosine kinases lead to decreased fibroblast activity.^5^, ^64^, ^65^ INPULSIS phase 3 trials I and II showed a significant decrease in the rate of FVC deterioration in IPF patients although the death rate remained the same.^66^ The side effects, primarily diarrhea and nausea, are manageable with medication, but hepatotoxicity may occur in rare cases and the drug is not recommended for those with severe liver disease.^67^, ^68^

Either nintedanib or pirfenidone are good choices as the primary treatment for IPF at the present time.^69^, ^70^, ^71^, ^72^, ^73^ The two drugs are able to slow deterioration in FVC and provide the patient with greater comfort for a noticeably longer time. However, use of these drugs may incur high out‐of‐pocket costs without changing the overall progression of the disease and the high mortality within 3 to 5 years after diagnosis.^74^ Some patients may not respond to these drugs, and in light of their expense and side effect profile, studies are underway to determine when these tyrosine kinase inhibitors may not be helpful.^75^

## 5 NONPHARMACOLOGIC THERAPY: LUNG TRANSPLANTATION

Lung transplantation may be a viable treatment option for IPF.^76^, ^77^ A key advantage compared with other modes of treatment is that it is the only method to improve both symptoms and survival time.^78^, ^79^ Previously, in the United States, COPD had been the main indication for lung transplant, but interstitial lung disease now supercedes COPD.^80^, ^81^

Determining the need for transplant is challenging, but in selecting IPF patients for transplant, risk of mortality and likelihood of survival after transplantation are key considerations. Furthermore, other comorbidities that may lead to complications such as cardiac dysfunction, GERD, diabetes, and obesity must be taken into account.^82^, ^83^, ^84^ Generally accepted benchmarks for lung transplant referral as well as contraindications to the procedure are based on guidelines proposed by the International Society of Heart and Lung Transplantation (ISHLT).^77^, ^85^ Among the key criteria for placement on the transplant list are rapid decline in FVC or DL_CO_, oxygen desaturation, or decrease in distance covered during 6‐min walk test, pulmonary hypertension, or hospitalization due to respiratory decline, pneumothorax, or acute exacerbation.

Importantly, telomere shortening and mutations in genes that control telomere function such as Telomerase Reverse Transcriptase (TERT) and Telomerase RNA Component (TERC) are associated with familial and sporadic cases of IPF and this may be considered in transplant decision‐making.^86^, ^87^, ^88^, ^89^ Although there is no conclusive evidence that transplant outcome is influenced by telomere length, short telomeres may confer vulnerability to bone marrow dysfunction upon administration of immunosuppressive drugs posttransplant.^90^, ^91^, ^92^ At this time, telomere length testing and genetic testing in IPF are not routine, but as more tests are performed, we may be able to better tailor immunosuppressants to the patient.^93^

Before transplant, antifibrotic treatment is generally maintained and strict adherence to medication regimen is encouraged, even as clinical trials are being conducted to determine if this affects transplant outcome.^94^, ^95^

Lung transplant may be either unilateral or bilateral. Benefits of unilateral transplant are the shorter wait times, easier procedure with lower perioperative complication rate, and the potential to improve the health of two patients from one donor.^90^ Data from the United Network for Organ Sharing database and the Organ Procurement and Transplantation network show a better long‐term survival rate for bilateral versus unilateral transplant.^96^, ^97^ A recent meta‐analysis found no survival advantage with bilateral lung transplant but did document better pulmonary function.^98^ Bilateral transplants can improve FVC and FEV significantly, and they can reach 100% of predicted values by 6–12 months. These measures also improve in unilateral transplant but reach about 80% of predicted values. Overall 5 year posttransplant survival is about 50%.

Follow‐up generally involves monitoring of FVC, DL_CO_, and pulmonary hypertension.^85^ Chronic lung allograft dysfunction and noncytomegalovirus infections are early causes of mortality.^99^ Other possible complications include immunosuppression, drug toxicity, infection, and neoplasia.^100^ Interestingly, a recent meta‐analysis found that several circulating tumor‐associated markers known to be elevated in IPF, including Ca125, Ca15.3, and Ca19.9, decreased significantly posttransplant.^101^

## 6 THE FUTURE: THERAPIES IN DEVELOPMENT

### 6.1 Stem cells

A nonpharmacological treatment being explored for used in IPF is based on the finding that mesenchymal stem cells (MSC), multipotent, undifferentiated cells, can regulate fibrotic processes and exert control over lung injury and repair.^102^, ^103^ MSC from IPF patients are dysfunctional and express reduced levels of tissue protective growth factors.^103^, ^104^, ^105^ In the Allogeneic human MSC in patients with IPF via intravenous delivery (AETHER) study, bone marrow‐derived MSC were infused intravenously into patients with IPF. AETHER was a nonrandomized, nonplacebo‐controlled phase 1 trial of a single MSC infusion. In this study, over the course of 6–12 months, the primary endpoint of safety was met.^106^, ^107^ A high cumulative dose stem cell infusion into patients with rapidly progressive IPF not taking antifibrotic drugs also showed excellent safety and tolerability.^108^ Human Autologous Lung Stem Cell Transplant for IPF (HALT‐IPF) (NCT04262167), a randomized trial of intravenous infusion of lung stem cells in persons with IPF, is currently enrolling.

### 6.2 Pentraxin

The pentraxin protein family consists of three highly phylogenetically conserved acute phase reactant plasma proteins made in the liver which are known to be involved in inflammation and innate immunity.^109^ Pentraxin 2 (PTX2, also known as serum amyloid P) is a constitutively synthesized member of the pentraxin protein family that can modulate wound healing and fibrotic remodeling of injured tissue.^110^ PTX2 acts to modify neutrophil adhesion and inhibit monocyte differentiation into profibrotic macrophages and fibrocytes.^111^, ^112^ It also binds to cellular debris, enhancing phagocytosis by leukocytes, and it inhibits the production of TGF‐β, a cytokine long considered a key fibrosis mediator.^113^, ^114^, ^115^ Serum PTX2 levels are significantly lower in IPF patients than in healthy controls.^114^ High local levels of PTX2 can delay wound healing via inhibition of fibrocytes and macrophages, which limits scarring and fibrosis around wounds.^116^ These antifibrotic properties are being explored for therapeutic benefits in fibrotic disorders such as IPF.^117^, ^118^, ^119^ The rationale for use of PTX2 in IPF is the clinical evidence that circulating fibrocyte concentrations (which correlate with abundance of fibroblastic foci in IPF tissue) above 5% of total blood leukocytes predict lifespan in IPF at an average of 7.5 months, while fibrocyte levels below 5% are associated with lifespan of about 27 months.^120^ Furthermore, bleomycin treatment induces prolonged lung inflammation and increased fibrosis in mice with genetic deletion of PTX2 compared with wild type mice.^121^

The first human clinical study on the pharmacokinetics and safety of PRM‐151, a recombinant human PTX2 protein, as a treatment for IPF, was a modest randomized, blinded, placebo‐controlled trial in 26 healthy volunteers between the ages of 18 and 53 years and 3 patients, 2 with familial interstitial pneumonia, and 1 with IPF, ages ranging between 29 and 72 years.^122^ The enrollees were given a single intravenous infusion varying from 0.1 to 20 mg/kg, and the drug was well‐tolerated with mild adverse effects in both placebo and treatment groups. No safety concerns were raised.

In a randomized, double blind, placebo‐controlled, multiple ascending dose trial of PRM‐151 conducted in 3 centers, 21 patients between the ages of 40 and 80 years diagnosed with IPF were given PRM‐151 in 5 intravenous infusions over 15 days at doses of 1, 5, or 10 mg/kg or placebo. All doses were well‐tolerated and raised circulating levels of PTX2. DL_CO_ was not affected by treatment, but 6‐min walking distance and FVC showed a trend toward an increase.^118^

In a second randomized, double‐blind placebo‐controlled phase 2 study (PRM‐151‐202) conducted at 18 sites in 7 countries, 117 patients with IPF between the ages of 40 and 80 years received placebo or 10 mg/kg PRM‐151 every 4 weeks for 24 weeks after a three‐dose loading regimen, along with usual IPF treatment in 78%. Compared with placebo, the mean percentage of predicted FVC and 6‐min walking distance declined significantly less in the PRM‐151 group.^123^

An open label extension study to week 76 in which 74 patients continued treatment with PRM‐151 and 37 placebo‐treated patients crossed over to receive the drug showed persistent positive effect of treatment in those who continued and slowed rate of decline of FVC in those who crossed over. As expected in an IPF population, adverse events accumulated over time and most were not deemed to be related to treatment.^124^ A phase 3 efficacy and safety study of PRM‐151 (NCT04552899) is in progress with estimated completion date of March 2023.

### 6.3 Pamrevlumab

Pamrevlumab is a fully human recombinant monoclonal antibody that targets connective tissue growth factor (CTGF) and thus can possibly limit fibrotic progression.^125^ It is being evaluated for use in locally advanced unresectable pancreatic cancer and in IPF.^126^ In PRAISE a randomized, double‐blind, placebo‐controlled phase 2 clinical trial, 103 IPF patients age 40 to 80 years were split into 2 groups, 50 received the drug, while 53 were given the placebo every 3 weeks for 48 weeks in total.^127^, ^128^ Of the original 103 patients, 78 (38 on pamrevlumab and 40 on placebo) completed the study. Pamrevlumab safely and effectively slowed lung function decline in IPF patients with mean change from baseline to week 48 in percentage of predicted FVC of −2.9% in the pamrevlumab group compared with a change of −7.2% in the placebo group. This represents a relative reduction in percentage of predicted FVC decline of 60.3% in the arm treated with pamrevlumab. Disease progression, measured as decline in percentage of predicted FVC ≥ 10%, or death, was lower in the pamrevlumab group (10.0%) than in the placebo group (31.4%), with a significant between‐group difference at week 48 (*p* = 0·013). Quantitative lung fibrosis scores obtained with high‐resolution computed tomography at 48 weeks showed significantly less fibrotic progression in the pamrevlumab group. The PRAISE findings are considered promising, but they are still limited by the small sample size. The ZEPHYRUS study (clinicaltrials.gov identifier NCT03955146), a phase 3 randomized, double‐blind, placebo‐controlled, multicenter clinical trial with plans to enroll 340 subjects, is ongoing and will evaluate efficacy and safety of pamrevlumab over 52 weeks.^129^, ^130^

### 6.4 Autotaxin inhibitors

Autotaxin (ATX) is an enzyme that hydrolyzes lysophosphatidyl choline into the signaling molecule lysophosphatidic acid (LPA).^131^, ^132^ ATX is expressed by bronchial epithelial cells and alveolar macrophages and is upregulated in IPF.^133^ It has been postulated that increased ATX generates more LPA in the lung, which then has profibrotic effects on epithelial cells, endothelial cells, and fibroblasts, thus contributing to IPF.^134^, ^135^ GLPG1690 is an orally available inhibitor of ATX, also known as lysophospholipase D. Based on this sequence of events, by inhibiting ATX, GLPG1690 may be of benefit in IPF. Initial studies in humans indicate that dosages of 200 and 600 mg per day of GLPG1690 will yield at least an 80% reduction in LPA in clinical trials.^136^ Two phase 3, randomized, placebo‐controlled trials of GLPG1690 have completed recruitment with a primary endpoint of rate of decline of FVC over 52 weeks.^137^ Unfortunately, in February 2021, the trials were discontinued due to the conclusion by the Independent Data Monitoring Committee that the benefit–risk profile did not support continuation.^138^ It is now uncertain whether other ATX inhibitors, such as BBT‐877, will continue to be evaluated as candidate treatments for IPF.^139^

### 6.5 Galectin‐3

Galectin‐3 is a beta‐galactoside‐binding protein with pro‐fibrotic properties that is present at elevated levels in bronchoalveolar lavage fluid from patients with IPF.^140^ TD139, a small molecule galectin‐3 inhibitor, was shown to be safe and well‐tolerated in IPF subjects and healthy controls in a phase 1/2 clinical trial (ClinicalTrials.gov Identifier: NCT02257177).^141^ In addition, IPF patients showed improvements in several plasma biomarkers of inflammation, including platelet‐derived growth factor and the chemokine CCL18. A phase 2b clinical trial (NCT03832946) with FVC as the primary outcome measure is now enrolling IPF patients.

### 6.6 Human antigen R

Human antigen R (HuR), a member of the Hu/embryonic lethal, abnormal vision family of RNA binding proteins, is known to promote excessive inflammation and fibrogenesis in murine models of diabetic cardiovascular disease and liver disease.^142^, ^143^ This has led to an interest in HuR as a contributing factor to IPF. Al‐Habeeb et al. recently found that levels of HuR are increased in the lungs of IPF patients and that TGFβ drives translocation of HuR from nucleus to cytoplasm in human lung fibroblasts. Knockdown of HuR in human lung fibroblasts mitigated induction of α‐smooth muscle actin protein as well as a number of extracellular matrix proteins by TGFβ. Further, HuR knockdown reduced TGFβ‐driven morphological changes indicative of myofibroblast differentiation.^144^ This link between HuR and myofibroblast transformation may indicate a potential new target for IPF treatment.

### 6.7 Cell‐based therapies

The IPF Cell Atlas is a major initiative designed to make available single‐cell sequencing data on multiple cell types present in the lung with the ability to compare control and IPF cell transcriptomes.^145^, ^146^ The creators of this website intend it to be used to achieve insight into pathogenesis that can lead to novel treatment approaches. The Atlas has already yielded information on a cell type enriched in the IPF lung known as aberrant basaloid cells.^145^ These cells express epithelial and basal cell markers as well as a cellular senescence signature and mesenchymal markers. They are found in distal lung parenchyma of IPF subjects in areas of fibroblastic foci and were also found in persons with a diagnosis of systemic sclerosis‐associated interstitial lung disease.^147^ It is hypothesized that these aberrant basaloid cells represent a common disease mechanism and that therapies may be directed toward this cell type in IPF and other fibrotic states in which they appear.

## 7 BIOMARKERS AND ASSESSING TREATMENT RESPONSE

Predicting the clinical course of IPF based on respiratory function and radiologic imaging is unreliable and, at this time, we cannot predict disease trajectory accurately.^148^, ^149^ Monitoring of IPF progression is most commonly accomplished by measuring change in FVC over 12 months, and a decline in FVC ≥ 10% is generally considered the threshold for affirming progression.^150^ There are no well‐established blood‐based biomarkers for IPF. Developing this type of biomarker for clinical use is a high priority that would be useful in early diagnosis and assessment of treatment efficacy. A number of possible blood biomarkers are being considered and evaluated. These include the receptor for advanced glycation end products (RAGE), which is highly expressed in alveolar epithelial type I cells and is significantly decreased in the lungs of patients with IPF.^151^ Decreased circulating RAGE levels correlate with declining lung function in IPF.^152^ Another possible biomarker assesses type I and III collagen turnover, which likely reflects the increase in interstitial collagen found in the lung in IPF. Measures of the serum levels of matrix metalloproteases that break down collagens type I and III using enzyme‐linked immunosorbent assays may lead to a clinically relevant IPF biomarker.^153^ As we try new IPF treatment approaches, the importance of accurate, reliable, reasonably priced assays to monitor the efficacy for clinical trial and real‐world application will be increasingly important.

## 8 CONCLUSION

There is an urgent need for more attention and in‐depth research into development of targeted treatments that prolong life and improve quality of life for persons with IPF.^154^ Pirfenidone and nintedanib are the two antifibrotic agents currently available for the treatment IPF. At this time, only lung transplant can alter its relentless course. Novel treatments under evaluation include pentraxin, pamrevulmab (monoclonal antibody against CTGF), and ATX inhibitors (Table 1). Also under study are agents that control TGFβ. One of these is PLN‐74809, a small molecule, dual selective inhibitor of the integrins αVβ1/αVβ6. These integrins are known to activate TGFβ. A phase 2a randomized, double‐blind, dose‐ranging, placebo‐controlled study of PLN‐74809 in IPF is ongoing (clinicaltrials.gov identifier NCT04396756). Another approach to targeting TGFβ is via silencing RNA as exemplified by TRK‐250, a single‐stranded oligonucleotide that produces siRNA targeting human TGFβ mRNA. A phase 1 study of this inhaled nucleic acid medication is in progress with an estimated completion date of April 2022 (clinicaltrials.gov identifier NCT03727802).

Gene and protein expression profiling is beginning to generate information on the mechanisms that result in lung damage in IPF.^155^, ^156^ Lipidomics are also being analyzed to expand our knowledge of how IPF progresses.^157^ The clinical and research communities must come together to find better ways to regulate fibrotic and inflammatory responses in the IPF lung.

## CONFLICT OF INTEREST

All authors have no conflict of interest to declare.

## ETHICS STATEMENT

No ethics approval was needed, no human subjects were involved in this review paper, and no consent to participate and publish was needed.

## AUTHOR CONTRIBUTIONS

Conception: Reiss, Glass. Literature review: Renna, Grossfeld, Agarwala. Figures and manuscript writing: Reiss, Glass, Spiegler, De Leon. All authors provided critical review of the manuscript and approved this draft.

## Data Availability

Data sharing is not applicable to this article as no datasets were generated or analyzed during the current study.
